# Emotional disorders among informal caregivers in the general population: target groups for prevention

**DOI:** 10.1186/s12888-015-0406-0

**Published:** 2015-02-15

**Authors:** Marlous Tuithof, Margreet ten Have, Saskia van Dorsselaer, Ron de Graaf

**Affiliations:** Netherlands Institute of Mental Health and Addiction, PO Box 725, 3500 AS Utrecht, the Netherlands

**Keywords:** Informal care, Mood and anxiety disorders, Risk indicators, General population study

## Abstract

**Background:**

There are indications that informal caregiving negatively impacts caregivers’ mental health, but this was hardly examined using diagnoses of mental disorders and most studies used convenience samples without including non-caregivers as reference group. We examine whether informal caregivers more often have any emotional disorder, i.e. mood or anxiety disorder, than non-caregivers. Identify key risk indicators for any emotional disorder among informal caregivers in the general population.

**Methods:**

Data were used from the second wave of the Netherlands Mental Health Survey and Incidence Study-2 (NEMESIS-2), a nationally representative face-to-face survey (n = 5,303; aged 21–68). Respondents were defined as informal caregiver when they provided unpaid care in the 12 months preceding the second wave to a family member, partner or friend who needed care because of physical or mental problems, or ageing. Twelve-month DSM-IV diagnoses of emotional disorders were assessed using the Composite International Diagnostic Interview 3.0. Key risk indicators were identified using the following aspects: prevalence, odds ratio, attributable risk proportion, and number needed to treat. Sociodemographic, caregiving-related and other characteristics were considered as risk indicators.

**Results:**

In the past year, 31.1% of the respondents provided informal care, which ranged in time spent (8 or more hours/week: 32.1%) and duration (longer than 1 year: 48.7%). Informal caregiving was not associated with having any 12-month emotional disorder. Among caregivers, giving care to a first-degree relative, partner or close friend and giving emotional support increased the risk for any emotional disorder. Moreover, using all aspects, target groups were identified for prevention: caregivers without a job, living without a partner, and with a lack of social support.

**Conclusions:**

Although informal caregivers do not have an increased risk of emotional disorders, key risk indicators were identified using four aspects. Especially informal caregivers with limited resources (unemployment, living without a partner, lack of social support) may benefit from targeted prevention whereas general prevention measures may be desirable for carers with a burdensome care situation (giving care to a close loved one or providing emotional support).

## Background

In most Western countries, the elderly population is growing and, due to age-related morbidity and disability, health care costs are rising [1-4]. However, as the current economic crisis forces cuts in health care expenditure, policy makers are seeking to promote community solutions, such as care and support from family and friends, i.e. informal care [5,6]. At present, approximately one third of the adult general population in Western countries provides informal care [7]. Due to projected demographic developments, these numbers may increase substantially in the future.

Previous research has shown that informal caregiving negatively impacts caregivers’ well-being as it affects their daily life and is associated with mental health problems such as depression (for overviews, see [8-11]). However, many studies were performed using convenience samples recruited via patients or service providers. Highly burdened caregivers are likely to be overrepresented in such studies. For example, in a study among informal caregivers of dementia patients, the average time spent giving care was almost 90 hours weekly, [12] whereas population-based research gives an average of 20 hours [7]. Another study among caregivers of depressed patients observed that 85% of the caregivers lived with the patient, [13] whereas this is only 25% in the general population [14]. Thus, findings based on convenience samples may overrepresent the negative consequences of caregiving [15,16]. Moreover, many studies did not include a control group (for overview, see [16]) and thus lacked a comparison with non-caregivers. Finally, whereas previous research observed that informal caregiving was associated with higher scores on screeners measuring depressed or anxious mood, [8,17-20] it is uncertain whether informal caregiving is similarly associated with diagnoses of mental disorders. To our knowledge, only one community-based study, conducted in 1990 in Ontario, examined the relationship between informal caregiving and mental disorders. In this study, about 15% of the adult population aged 15–64 provided informal care and a slightly increased risk of mental disorders was observed among these caregivers [21].

An emotional disorder, i.e. mood or anxiety disorder, may result in substantial consequences in terms of the quality of life of the caregiver [18] and quality of care is likely to be affected. Application of existing measures to prevent emotional disorders among informal caregivers (e.g. psycho-educational interventions or counseling [22]) is therefore important and general practitioners have been targeted as a critical first point of contact to identify the impact of caregiving [23]. However, as the group of informal caregivers is substantial, [7] with only a minority having an emotional disorder, [21] it may be efficient to target prevention measures to those at increased risk of emotional disorders [14,24]. Identification of risk indicators facilitates such selection. Ideally, risks indicators should 1) identify a small and feasible target group; 2) be strongly associated with emotional disorders among informal caregivers; 3) result in a substantial health gain at population level if their adverse effect is blocked; and 4) require a low number of people who need an intervention to prevent one case of emotional disorder [24]. Most previous research on diminished mental health among informal caregiving focused on the second aspect. Based on these findings, a close relationship with the care recipient, [14,25,26] more hours of caregiving [27] and living with the care recipient [12,28] as well as general characteristics such as employment, [28,29] or lack of social support [17,30-32] can be identified as potential risk indicators for emotional disorders among informal caregivers. Moreover, time-consuming activities, such as work or having children at home, in combination with many hours of caregiving may result in particularly high risks of adverse outcomes [29].

Using data from the second wave of the Netherlands Mental Health Survey and Incidence Study-2 (NEMESIS-2), a psychiatric epidemiological study among the Dutch general population aged 21 to 68 years at that wave, we examined i) whether informal caregiving is associated with presence of any emotional disorder in the past year; and ii) which characteristics (i.e. sociodemographic, caregiving-related and other characteristics) are risk indicators for any emotional disorder among informal caregivers.

## Methods

NEMESIS-2 is a psychiatric epidemiological cohort study of the Dutch general population. It is based on a multistage, stratified random sampling of households. Based on the most recent birthday at first contact within the household, an individual aged 18–64 years with sufficient fluency in the Dutch language was randomly selected. The study was approved by the Medical Ethics Review Committee for Institutions on Mental Health Care (METIGG). After having been informed about the study aims, respondents provided written informed consent. A comprehensive description of the design is provided in [33]. Informal caregiving was assessed during the second wave and therefore data from this wave were used for the present study.

In the first wave (T_0_), performed from November 2007 to July 2009, a total of 6,646 persons aged 18–64 were interviewed (response rate 65.1%; mean duration of 95 minutes). This sample was nationally representative, although younger subjects were somewhat underrepresented [33]. All T_0_ respondents were approached for follow-up (T_1_), three years after T_0_ from November 2010 to June 2012. Of this group, 5,303 persons were interviewed again (response rate 80.4%, with those deceased excluded). Attrition was not significantly related to any 12-month disorder, any anxiety, any mood, or any substance use disorder, nor to the separate disorders, after controlling for sociodemographics [34]. The mean duration of the second interview was 84 minutes. The mean period between both interviews was 3 years and 7 days (1,102 days; standard deviation = 64; ranging from 2 to 4 years).

The face-to-face interviews were mainly held at the respondent’s home and were conducted by trained professional interviewers (65 at the second wave) of the fieldwork agency GfK (Growth from Knowledge) Panel Services Benelux, with their team of five supervisors. Interviewers were selected on their experience with systematic face-to-face data collection, experience with sensitive topics and ability to achieve a good response in other studies. Fieldwork was monitored over the entire data collection period by the NEMESIS-investigators and the fieldwork agency (for more information on quality checks of the data, see [33]).

### Informal caregiving and caregiving-related characteristics

Respondents were defined as informal caregiver when they provided unpaid care in the 12 months preceding the second wave to a family member, partner or friend who needed care because of physical or mental problems, or ageing (n = 1,759). Follow-up questions measured to whom they gave care (first-degree family such as parents, siblings or children; other family such as in-laws or other; partner; friend), whether they lived with the care recipient, reasons for care (physical problems; mental problems; ageing; dementia), kind of care (domestic work; personal care or nursing; emotional support or supervision; practical care), time spent giving care (0–7 hours weekly; 8 or more hours weekly) and duration of care (less than 1 year; 1 year or longer).

### Emotional disorders

DSM-IV diagnoses [35] were made using the Composite International Diagnostic Interview (CIDI) 3.0, a fully structured lay-administered diagnostic interview. This instrument was developed and adapted for use in the World Mental Health Survey Initiative [36] The CIDI 3.0 version used in NEMESIS-2 was an improvement of the Dutch one used in this initiative. The disorders considered in this paper include mood (major depression; dysthymia; bipolar disorder) and anxiety disorders (panic disorder; agoraphobia without panic disorder; social phobia; generalized anxiety disorder) in the past 12 months, assessed at the second wave. Clinical calibration studies in various countries [37] found that the CIDI 3.0 assesses mood and anxiety disorders with generally good validity in comparison to blinded clinical reappraisal interviews. For the present study, mood and anxiety disorder were combined into presence or absence of any emotional disorder.

### Sociodemographics

Age, gender, educational level (primary, basic vocational or lower secondary education vs higher education), employment status, partner status and having children living at home (vs having children not living at home and having no children) were considered. Age, gender, and educational level were measured at T_0_, other sociodemographics were measured at T_1_.

### Other characteristics

Social support from three resources (partner; family or friends; neighbors) was measured with two questions regarding the extent respondents could rely on the resource for help and could open up to them (four response categories ranging from “not at all” to “a lot”). The mean score on the two questions was used to indicate social support perceived from a particular resource, taking the respondents’ evaluation of it into account. The total social support measure (ranging from 1 to 4) was calculated as the mean score on the perceived social support from at least two resources, because not all respondents had a partner [38]. A dichotomous variable was constructed using a cut-off point of 2.5 (the middle of the scale), thereby separating the lowest twelve percent, a group with quite severe lack of social support, versus the rest. Sensitivity analyses with cut-off points of 2 (4%) and 3 (31%) resulted in similar findings.

A standard checklist assessed presence of 17 chronic physical disorders (asthma; chronic obstructive pulmonary disease; chronic bronchitis; emphysema; severe heart disease; heart attack; hypertension; stroke; stomach or intestinal ulcers; severe intestinal disorders; diabetes; thyroid disorder; chronic back pain; arthritis; migraine; impaired vision or hearing; and another chronic physical disorder), treated or monitored by a medical doctor in the prior 12-months [39].

### Analyses

Analyses were performed using Stata version 12.1, [40] which enabled to control for the complex sampling and recruitment procedure of the study. The data were weighted to correct for differences in the response rates in several sociodemographic groups at both waves and differences in the probability of selection of respondents within households at baseline.

Using the complete sample (n = 5,303), characteristics of informal caregiving were examined using frequencies and logistic regression analyses adjusted for age and gender. The association between informal caregiving and presence of 12-month emotional disorder was examined with logistic regression analyses adjusted for all sociodemographics. Sensitivity analyses were performed with stricter definitions of informal care; i.e. eight or more hours weekly, longer than one year, or both.

Further analyses were conducted among the informal caregivers (n = 1,759). That is, potential risk indicators of emotional disorders among informal caregivers were first separately investigated with bivariate logistic regression analyses adjusted for age and gender, and then simultaneously with conventional backstepping procedures to obtain a parsimonious set of risk indicators. No collinearity of risk indicators was observed in the final model. Lastly, the Population Attributable Risk Proportion (PARP) and the Number Needed to Treat (NNT) of the final set of significant risk indicators were calculated in univariable analyses to select useful indicators for targeted prevention [24]. The PARP represents the proportion of emotional disorders in the population of informal caregivers that can be reduced when the adverse effect of the risk indicator is completely eliminated [41]. Although this assumption is not realistic as preventive programs are often unable to completely block the adverse effect of the risk indicator and some risk indicators are unmodifiable (i.e. age, gender), the PARP can be useful as it gives an indication of the highest achievable health gain. The PARP was examined using the punaf procedure of Stata [42]. The NNT indicates how many informal caregivers have to receive an intervention to avoid one case of emotional disorder, assuming that the adverse effect of the risk indicator is completely blocked by the intervention [24,43]. The NNT can best be interpreted as a minimum of the required effort, as interventions are rarely completely effective. This was calculated as the inverse of the risk difference between informal caregivers with and without the risk indicator, which was estimated with generalized linear models with a binomial distribution and an identity link function.

## Results

### Characteristics of informal caregivers

About one third (31.1%) of the respondents provided unpaid care to a family member or friend in the 12 months preceding the interview. Informal caregivers were more often female, 45 or older and had a higher educational level than non-caregivers (Table 1). Notably, informal caregivers did not differ from non-caregivers with regard to employment status, partner status, having children at home, lack of social support or having a chronic physical disorder.

**Table 1:** **Characteristics of informal caregivers**

	**Non-caregiver (** ***n*** **= 3,544; 68.9%)**	**Informal caregiver (** ***n*** **= 1,759; 31.1%)**		
	**%** ^**a**^	**%** ^**a**^	**OR** ^**b**^	**95% CI** ^**b**^
***Sociodemographics***				
Female gender	45.0	59.6	1.84***	1.59-2.12
Younger than 45	54.7	36.2	0.46***	0.41-0.53
Low educational level	30.2	28.0	0.81*	0.69-0.95
Unemployed	22.7	29.5	1.06	0.89-1.26
Living without a partner	31.8	25.9	0.83	0.69-1.00
Children at home	45.7	44.0	0.99	0.87-1.13
***Other characteristics***				
Lack of social support	11.8	11.2	0.87	0.69-1.09
Chronic physical disorder	37.1	45.5	1.09	0.93-1.28

*p < 0.05; ***p < 0.001; OR: Odds Ratio; CI: Confidence Intervals.

^a^Column percentages.

^b^Logistic regression models, adjusted for age and gender.

### The association between informal caregiving and having a 12-month emotional disorder

The prevalence of any emotional disorder was similar among informal caregivers and non-caregivers (7.5% vs 8.8%; p = 0.15). Logistic regression analysis adjusted for all sociodemographics (not in table) showed no association between informal caregiving and having an emotional disorder (OR = 0.86; 95% CI = 0.66-1.11). When informal caregiving was defined more strictly, as giving care eight or more hours weekly, longer than one year, or both, no association with an emotional disorder was observed (OR = 0.92; 95% CI = 0.64-1.31, OR = 1.22; 95% CI = 0.92-1.63 and OR = 1.17; 95% CI = 0.73-1.87, respectively).

### Risk indicators for 12-month emotional disorder among informal caregivers

More than half of the respondents provided care to a first-degree family member (first column of Table 2), almost one third to other family members, 12% to their partner and 16% to a friend. Approximately 40% of caregivers provided care to more than one person and 17% lived with the care recipient. Physical problems were the most common reason for giving care (61%). Almost all informal caregivers provided emotional support (89.5%) or practical care (77.3%). About one third provided eight or more hours of care weekly, and about half of caregivers provided care for one year or longer.

**Table 2:** **Risk indicators of 12-month emotional disorder among informal caregivers (n = 1,759)**

		**Model 1** ^**a**^		**Model 2** ^**b**^			
	**%**	**OR**	**95% CI**	**OR**	**95% CI**	**PARP**	**NNT**
***Sociodemographics***							
Female gender	59.6	2.05**	1.20-3.50	1.68*	1.00-2.82	37.3	21.2
Younger than 45	36.2	2.00**	1.24-3.24	2.53**	1.44-4.43	25.3	19.0
Low educational level	28.0	1.14	0.68-1.89				
Unemployed	29.5	2.67***	1.56-4.55	2.23**	1.29-3.87	25.7	15.3
Living without a partner	25.9	2.34***	1.51-3.65	1.87**	1.20-2.91	26.6	13.0
Children at home	44.0	0.72	0.47-1.11				
***Caregiving characteristics***							
Care recipient							
First-degree family	55.3	1.33	0.88-2.01	2.04**	1.28-3.25	12.7	57.9
Other family	30.0	0.35***	0.20-0.61				
Partner	11.8	1.54	0.82-2.88	2.17*	1.10-4.28	3.2	49.1
Friend	16.4	1.92**	1.21-3.07	3.46***	1.73-6.92	13.0	16.9
More than one care recipient	39.0	0.81	0.54-1.20	0.53*	0.30-0.96	-	-
Living with care recipient	16.6	1.40	0.85-2.30				
Reason for care							
Physical problems	61.2	0.71	0.48-1.06				
Mental problems	29.9	1.87**	1.26-2.79				
Ageing	30.7	0.63	0.37-1.07				
Dementia	12.2	1.44	0.85-2.45				
Kind of care							
Domestic work	59.1	1.19	0.77-1.82				
Personal care or nursing	27.0	1.39	0.89-2.16				
Emotional support	89.5	6.83***	2.49-18.76	6.16***	2.18-17.39	83.0	14.4
Practical care	77.3	1.45	0.82-2.57				
Eight or more hours per week	32.1	1.19	0.78-1.79				
Longer than one year	48.7	1.88**	1.20-2.93	1.92**	1.19-3.12	22.6	28.6
***Other characteristics***							
Lack of social support	11.2	4.55***	2.80-7.40	3.22***	1.97-5.25	23.7	6.3
Chronic physical disorder	45.5	1.52	0.97-2.38				

*p < 0.05; **p < 0.01; ***p < 0.001; OR: Odds Ratio; CI: Confidence Intervals. PARP: Population Attributable Risk Proportion; NNT: Number Needed to Treat.

^a^Logistic regression models, adjusted for age and gender.

^b^Final backward stepwise multivariable logistic regression model.

Results for the two types of regression models, i.e. bivariate analyses adjusted for age and gender and the final backstep model, are shown in Table 2. Notably, findings are largely similar for both types of models. Therefore, hereafter we will describe the results of the most parsimonious model, the final backstep model. Regarding sociodemographics, emotional disorder among informal caregivers was associated with female gender, being younger than 45, unemployment and living without a partner, but not with educational level and having children at home. Of the caregiving characteristics, giving care to a direct family member, partner or close friend, providing emotional support, and giving care for one year or longer were associated with an emotional disorder. Unexpectedly, providing care to more than one care recipient was associated with a lower chance of an emotional disorder. Living with the care recipient, reason for care, and giving eight or more hours of care weekly were not associated with an emotional disorder. Moreover, neither employment status nor having children at home strengthened the relationship between hours of care provided and having an emotional disorder, that is, no additive interaction-effects were observed (not in table) [44]. Finally, caregivers who perceived a lack of social support had higher chances of experiencing an emotional disorder. No association between chronic physical disorders and emotional disorder was observed.

In the final two columns of Table 2, the PARP and the NNT are displayed for the risk indicators in the final backstep model. Emotional support had the highest PARP (83.0%) and an acceptable NNT (14.4), but the high prevalence rate (89.5%) limits its use for targeted prevention. Another notable risk indicator is lack of social support, with a substantial PARP (23.7%), the lowest NNT (6.3) and a fairly low prevalence rate (11.2%). Finally, being unemployed and living without a partner had considerable PARPs (25.7% and 26.6%, respectively), relatively low NNTs (15.3 and 13.0) and manageable prevalence rates (29.5% and 25.9%).

## Discussion

### Key findings

In line with previous cross-national research, [7] we observed that informal caregiving was quite common (31.3%) in the general population. Although no association between informal caregiving and emotional disorders was observed, risk indicators were identified. Specifically, informal caregivers with limited resources (lack of social support, unemployment, living without a partner) and those with a burdensome care situation (giving care to a close loved one or providing emotional support) were at higher risk of an emotional disorder.

### Strengths and limitations

The use of a large population-based sample which included different types of informal caregivers, not limited to heavily burdened caregivers, as well as a reference group of non-caregivers, and the use of diagnoses measured with a valid instrument (CIDI 3.0), are important strengths of our study. To our knowledge, this is the first study to examine risk indicators for emotional disorders in a representative sample of informal caregivers and to identify indicators useful for prevention activities.

A few cautionary remarks should be made in relation to our findings. Although the NEMESIS-2 sample was representative of the Dutch population on most parameters, people with an insufficient mastery of the Dutch language were underrepresented. Moreover, although NEMESIS-2 had a broad age range, the maximum age of the sample at the second wave was 68, thereby excluding older caregivers. Hence, our findings are not generalizable to older age groups. Furthermore, non-response may have been higher among heavily burdened caregivers with too little time to participate. The extent to which this occurred is unclear. Another limitation is that only the second wave of NEMESIS-2 could be used and our findings were therefore based on cross-sectional data only. One should thus be careful in interpreting these findings as no causal relationships were examined and reverse-causality may also play a role. That is, an emotional disorder may contribute to presence of the risk indicators.

### Findings

Informal caregiving was quite common in the Dutch general population (31.3%). Informal caregivers were more often female, 45 years or older, and had a higher educational level than non-caregivers. Caregivers and non-caregivers did not differ in employment status, partner status, having children at home, social support and presence of a chronic physical disorder.

Providing informal care was not significantly associated with having an emotional disorder and even a lower prevalence of emotional disorder was found among caregivers compared to non-caregivers. In this regard, our findings are at odds with findings from a similar population-based study performed in Canada in 1990, in which informal caregivers were found to have a slightly higher chance of mood and anxiety disorders than non-caregivers. Although that study used a similar age range and the same definition of informal care, [21] the percentage of informal caregivers in the population was much smaller (15%) than in our study (31%). As that study used the same diagnostic instrument to examine mental disorders (though an earlier version), the discrepancy is likely due to societal changes over time (giving informal care might be more common nowadays) or cross-national differences. The fact that we did not observe an association between informal caregiving and emotional disorder, even when stricter definitions of informal care were applied (eight or more hours weekly, longer than one year, or both), might be explained by a continuing selection process, such that those who provide informal care tend to be healthier than those who either do not start or quit caregiving. A similar phenomenon is seen in the workforce, with a lower prevalence of mental disorders among workers than those outside the workforce, [39] dubbed the ‘healthy worker effect’ [45]. Such a phenomenon thwarts examination of the relationship between informal caregiving and emotional disorder in cross-sectional research.

Despite major methodological differences, findings regarding risk indicators of emotional disorders among informal caregivers are largely in line with previous research primarily based on convenience samples [15,16]. Overall, emotional disorders among informal caregivers seem to be related to having limited resources: lack of social support was a strong indicator for emotional disorders among informal caregivers, [17,30-32] as well as unemployment and living alone [46]. Other significant sociodemographic correlates for an emotional disorder were being female and younger age, but not educational level [12,24,46]. Although most of these risk indicators also exist in the general population, [47] they help to identify vulnerable informal caregivers.

Among informal caregivers, duration of care was associated with an emotional disorder, but not time spent giving care. The latter finding was in contrast with previous research [14] and might be explained by positive aspects associated with caregiving, such as increased closeness with the care recipient and personal satisfaction [46,48,49] Such aspects can positively impact mental health, provided that caregiving activities are not too burdensome [46] Our findings could indicate that the positive effect of such rewards offset the burden of much time spent giving care, but does not overcome the burden of a long duration of care. Future research should examine this point. Although a meta-analysis observed a poorer outcome for predominantly spousal caregivers of dementia patients compared with other diseases, [8] we did not observe such a relationship. Although power limitations cannot be excluded, as only 12% of our sample provided care to a dementia patient, there is a more likely explanation: our sample was relatively young and the majority of caregivers of dementia patients provided care to a parent, rather than to a partner. As the relationship with the care recipient in combination with a specific illness may affect the outcome for caregivers, [25] sampling differences (predominantly spousal caregivers vs all types of caregivers) may explain the difference in findings.

Some other caregiving-related indicators are worth noting. Specifically, giving care to a close loved one, i.e. a first-degree relative, partner or close friend, put informal caregivers at higher risk for an emotional disorder [14]. This might be because in such cases the illness of the care recipient is also burdensome. A previous study observed that giving care to a parent was associated with poorer mental health, but giving care to an in-law was not [26]. It was suggested that this difference is due to type of care provided, with less emotional support and more practical care [26]. Similarly, we observed a higher risk of emotional disorder when the caregiver provided emotional support.

Our findings suggest that tailoring prevention measures to people with limited resources (lack of social support, unemployment, living without a partner) can result in health gain (PARP) with relatively low effort (prevalence and NNT). Low-level interventions, such as e-health interventions [50] at the first signs of distress, may suffice to prevent an escalation of problems. As informal caregivers may join the care recipient when visiting the general practitioner (GP) or consult the GP when first signs of distress appear, GPs could play a key role in identifying caregivers at risk of emotional disorders. They should be particularly attentive when caregivers have limited resources. Close and more intense care situations (giving care to a close loved one or giving emotional support) may also be worthy of their attention. However, as such care situations are common, a more general approach may be appropriate, for example providing extra coping tools on websites targeted to informal caregivers.

## Conclusions

Findings from this population-based study show that informal care is frequently provided in the general population, but varies significantly in terms of time spent, duration, and type, and is not necessarily associated with a higher chance of emotional disorders. Although this seems to contradict the widely accepted notion that informal caring negatively affects well-being, [51,52] it may also show that findings for convenience samples are not applicable to informal caregiving in the general population. Moreover, informal caregivers who continue giving care may be healthier than those who do not start or quit caregiving. The risk indicators identified here, such as limited resources, giving care to a close loved one, and giving emotional support, could help to target prevention measures to informal caregivers at risk for emotional disorders.
